# Comparison of Serum Cystatin C and Creatinine Levels among Individuals with Persisting Proteinuria in Farming Communities of Rural Sri Lanka

**DOI:** 10.21315/mjms2018.25.6.7

**Published:** 2018-12-28

**Authors:** Jayasekara Mudiyanselage Kithsiri Bandara Jayasekara, Dhammika Maneke Dissanayake, Fathima Shihana, Ramaiya Sivakanesan, Rajith Niloshan Silva, Suwanda Hennadige Nandana Priyankara Gunawickrama

**Affiliations:** 1 Department of Medical Laboratory Sciences, Faculty of Allied Health Sciences, Sir John Kotelawala Defence University, Sri Lanka; 2 Department of Pathology Faculty of Medicine, University of Peradeniya, Sri Lanka; 3 South Asian Clinical Toxicology Research Collaboration, Faculty of Medicine, University of Peradeniya, Sri Lanka; 4 Department of Biochemistry, Faculty of Medicine, University of Peradeniya, Sri Lanka; 5 Institute for Combinatorial Advanced Research and Education, General Sir John Kotelawala Defence University, Ratmalana, Sri Lanka

**Keywords:** persistent proteinuria, cystatin C, chronic kidney disease of uncertain aetiology, Sri Lanka

## Abstract

**Background:**

Chronic kidney disease of uncertain aetiology (CKDu) is one of the major health concerns among agricultural communities in Sri Lanka. Individuals involved in severe agricultural works for their livelihood are highly vulnerable for this disease and patients have been detected with persisting proteinuria at community-level screening. The current study was designed to evaluate the diagnosis of two functional markers of kidney damage using individuals with persisting proteinuria as the baseline.

**Methods:**

One hundred and fifty hard-working agricultural farmers from high-prevalence area for CKDu (Madawachchiya) were screened three times for proteinuria; 66 proteinuric and 21 non-proteinuric were identified as the baseline classification. Selected individuals were analysed further for creatinine, protein and cystatin C in urine and creatinine, cystatin C in serum. Urine protein-to-creatinine ratio (UP/UC) was calculated.

**Results:**

Based on creatinine and cystatin C cut-off levels in serum, individuals were classified as high or normal. Diagnosis of two functional markers (creatinine and cystatin C) were evaluated using receiver operating characteristic (ROC) curve and in terms of sensitivity and specificity using UP/UC as the baseline. Creatinine and cystatin C-based eGFR (estimated Glomerular filtration rate) levels were calculated, and Pearson’s correlation coefficient was determined between different eGFR measurements using UP/UC. Mean (SD) UP/UC ratio, serum creatinine, and serum cystatin C levels of the proteinuric subjects were 129.0 (18.4) mg/mmol, 1.35 (0.39) mg/dL, 1.69 (0.58) mg/L. For non-proteniuric individuals, the results were found to be 14.4 (2.28), 1.22 (0.40) mg/dL, 0.82 (0.25) mg/L. The ROC analysis showed excellent accuracy in using cystatin C for identifying proteinuric patients than creatinine area under the curve (AUC): 0.9675, *P* < 0.001). Cut-off points were identified as 1.015 mg/dL for serum creatinine and 0.930mg/L for cystatin C. Furthermore, cystatin C based Hoek formula showed the better correlation (0.635, *P* < 0.001) with UP/UC compared with creatinine based modification of diet in renal disease (MDRD) formula.

**Conclusion:**

The study showed elevated serum cystatin C in patients with persisting proteinuria compared with non-responding serum creatinine. Moreover, cystatin C-based eGFR equations were more accurate to determine the kidney function than serum creatinine in proteinuric patients who are vulnerable for CKDu in high-prevalence areas.

## Introduction

Chronic kidney disease (CKD) is a public health problem with a prevalence of about 10% in many populations worldwide (1). According to the Kidney Disease Outcomes Quality Initiative (KDOQI) Clinical Practice Guidelines, all individuals should be evaluated at first encounter to determine whether they are at increased risk of developing CKD (2). Ascertainment of risk factors should be done by considering patients’ sociodemographic, past health records and family history. Moreover, blood pressure, and blood glucose or HbA1C levels enable the clinician to determine whether a patient is at increased risk of this disease. Identified individuals should be evaluated further (2, 3). Clinical evaluation of patients at increased CKD risk encompasses measurements of blood pressure, serum creatinine to estimate glomerular filtration rate (GFR), and the protein or albumin to creatinine ratio in urine. Examination of urine sediments for erythrocytes and white blood cells is also part of the examination. Beyond screening approaches, patients at high risk should be evaluated by ultrasound imaging, urine-specific gravity or osmolality, serum electrolytes and kidney biopsies (2, 4).

The KDOQI proposes that CKD diagnosis, staging and monitoring should be based on GFR estimates (2). Several formulae have been developed based on serum creatinine, such as modification of diet in renal disease (MDRD) and Cockcroft-Gault (CG) equations. However, serum creatinine is an insensitive marker of kidney injury for two reasons (5). First, there is a broad reference range because creatinine production differentiates among variable muscle mass. Individuals who are involved in extensive muscle labour on the day before testing are likely to render unreliable results. This is true among farmers. Secondly, the inverse relationship between GFR and serum creatinine is such that large GFR declines are caused by small rises in serum creatinine (6). Notably in this regard, in diminutive or elderly (age > 60 years) people with small muscle mass, serum creatinine will remain within the reference limits despite substantial kidney damage (7).

Measurements of urinary creatinine does not provide a solution as the results are often inaccurate due to tubular creatinine secretion and by errors in specimen collection (6). Measurements of creatinine clearance can be useful in patients on a vegetarian diet or with low muscle mass. Renal clearance of infused ‘tracers’ such as inulin, iothalamate or iohexol, can provide accurate measurements of GFR, but they are expensive and inappropriate for routine clinical use and screening (8).

The need for dependable markers of impaired renal function or injury is clear. In this context, cystatin C (9), a small plasma protein of ~13 kDa that inhibits cysteine proteases (7), remains an alternative. It is produced by all nucleated cells and is small enough to filter itself out of the blood at the glomerulus. The serum cystatin C concentration correlates inversely with GFR, and assays for cystatin C are commercially available. The production rate of cystatin C was initially claimed to be constant, although recent studies have detected decreased cystatin C production in transplant patients with low GFR (10, 11). Although this may hinder the interpretation of cystatin C concentrations under advanced renal failure, it may not limit the use of cystatin C measurements to detect early renal dysfunction. Other low-molecular-weight proteins and a glyco-conjugate of tryptophan (12) have also been tested as markers of renal function, but they have not been investigated thoroughly.

CKDu is widely spread in the North Central Region (NCR) of Sri Lanka. Epidemiological characteristics explained that 92% of the patients are farmers. According to medical statistics, over 150,000 patients have been treated (13) and the age-adjusted prevalence is increasing from 30–40 years, it was over 20% in people aged over 50 years. Five main high prevalence clusters for CKDu have been identified, namely Madawachchiya, Padaviya, Kabithigollawa, Elahara and Girandurukotte (13).

Farmers aged over 30 years, involved in severe agricultural activities for more than five years, cultivating more than two hectares of land, using agrochemicals and having family history of CKDu were identified as risk factors (14, 15). CKDu was initially identified by measuring persisting proteinuria (urine dipstick protein positive on two consecutive occasions) at community level screening programmes (16). Urine protein-to-creatinine ratio is used to confirm dipstick and kidney function is determined by creatinine-based eGFR. However, histopathological research conducted on asymptomatic patients revealed that 27% and 50% of the patients were in stage 1 and stage 3, respectively (17). Therefore, the current study was designed to compare the serum creatinine-based eGFR measurements with cystatin C as a promising marker for identifying the level of kidney function using the UP/UC ratio as the base line.

## Materials and Methods

Five high-prevalence areas (clusters) for CKDu were identified in Sri Lanka and one area, Madawachchiya, was selected randomly for the study. The prevalence of CKDu in this area was around 10% and the sample size was calculated to indicate a size of 138 subjects. Therefore, an estimate of 150 individuals were selected and recruited for the study, they were chosen randomly from three selected villages in the Medawachchiya area. The study was conducted throughout a 6-month period starting from November 2013 to July 2014, covering the main cultivating season (Maha season) of paddy-farming communities. Small clusters of individuals who were working on the paddy for land preparations and other extensive works were recruited. According to the risk factors for the disease, farmers aged between 30–60 years, involved in agricultural activities for more than five years, cultivating more than two hectares of land, frequently using agrochemicals and having family history of CKDu were selected. Currently diagnosed CKD patients and patients with other chronic and significant illnesses (medically reported) such as diabetes mellitus, were excluded from the study.

Urine protein, urine creatinine and urine protein to creatinine ratio (turbidimetric assay, accuracy 2mg/dL and Jaffe rate-blanked and compensated for the Hitachi System) were determined in three early morning urine samples at two-week intervals. Sixty-six individuals with persistent proteinuria (> 15 mg/mmol) of creatinine at all three measurements were identified as proteinuric patients and 21 identified as non-proteinuric individuals (< 15 mg/mmol) of creatinine in all three measurements. The study group thus comprised of 87 subjects, and other 58 individuals were excluded.

Whole blood (5 mL) was collected from each of the 87 identified subjects. Blood was centrifuged within 1 h of collection and serum was stored at 20 °C for 1 day. Creatinine in serum was analysed using picric acid (Roche reagents, Jaffe rate-blanked and compensated for Hitachi 912 System). Serum cystatin C was estimated by Human sandwich ELISA method. Urine protein to creatinine ratio (UP/UC) of each individual was calculated (2, 18) and used as a criteria of identifying proteinuric patients. Urine protein to creatinine ratio greater than 15 mg of protein to mmol of creatinine was considered proteinuric and less as normal individuals (2).

Upper limits of the serum creatinine and cystatin C levels of all individuals were determined according to the gender specific normal ranges of the test kits. The differences of two functional markers as serum creatinine and cystatin C were compared in proteinuric and non-proteinurin individuals with students’ *t*-test. Further individuals were classified as patients (test positive) or as normal (test negative) for both serum creatinine and cystatin C. Serum creatinine greater than 1.2 mg/dL and 1.1 mg/dL for males and females, respectively were considered as test positive (2). Serum cystatin C levels greater than 1.0 mg/L for both males and females were considered as test-positive. True positive, false positive, true negative and false negative individuals were identified separately with relation to the proteinuria-based diagnosis (proteinuric or non-proteinuric). The sensitivity, specificity, maximum likelihood ratios and position of cut-point values were analysed by using Receiver Operating Characteristic (ROC) curves compared to UP/UC ratio.

Estimated GFR was calculated with serum creatinine levels of both proteinuric patients and normal individuals using MDRD formula:

$$\text{eGFR }(\text{mL}/\text{min}/1.73\;{\text{m}}^{2})=186.3\times (\text{serum creatinine mg}/{\text{dL})}^{-1.154}\times {\left(\text{age}\right)}^{-0.203}\times 0.742\;(\text{if female}).$$

eGFR values were also calculated using the following formulae with serum cystatin C (19, 7),

1. Hoek eGFR = −4.32 + 80.35 × L/cystatin C in mg/L
2. Larsson eGFR = 77.239 × cystatin C in mg/L^−1.2623^

The relationships among the above calculations were tested with UP/UC ratio in terms of Pearson correlation co-efficient. The study was approved by the Ethical Review Committee, Faculty of Medicine, University of Peradeniya, Sri Lanka the Provincial Director (Health Services), Anuradhapura, Sri Lanka.

## Results

There were 53 males and 34 females in the study group with the mean (SD) age of 47 (12). According to the KDOQI guidelines (2), based on the UP/UC ratio, 66 subjects were identified as proteinuric and 21 as non-proteinuric. Mean UP/UC ratios were not significantly different (*P* = 0.163) between male and female subjects.

Significant differences were noticed in the cystatin C levels of proteinuric individuals with non-proteinuric individuals in both males and females (*P* = 0.038). However, the serum creatinine levels of two groups were similar (*P* = 0.282) between the genders (Table 2).

**Figure 1:**
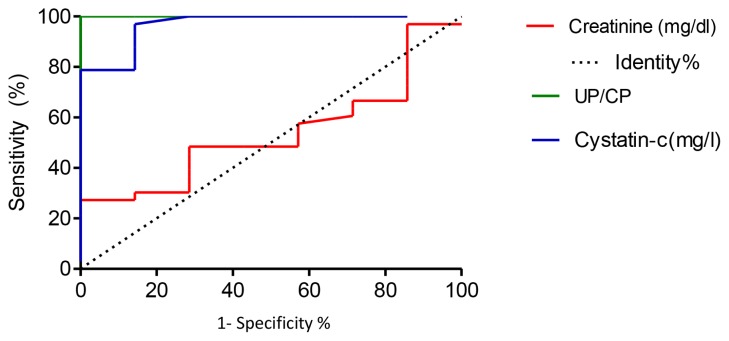
ROC of serum creatinine and cystatin C for identifying the proteinuric patients

Individuals identified as proteinuric and non-proteinuric (Table 1) were further divided based on their serum creatinine and cystatin C. Serum creatinine greater than 1.2 mg/dL and 1.1 mg/dL for males and females, respectively, was considered as test positive. Serum cystatin C levels greater than 1.0 mg/L for both males and females were identified as test positive. Identified individuals based on the proteinuria, sensitivity and specificity of serum creatinine and serum cystatin C were analysed using ROC curves (Table 3). According to the ROC analysis (Figure 1), cystatin C showed excellent correlation with UP/UC compared with serum creatinine for identifying proteinuric patients (2).

No significant difference was observed between mean eGFR (MDRD) levels of proteinuric patients and non-proteinuric individuals in the creatinine-based formula (Table 4). However, significant decreases of eGFR were noticed (*P* < 0.05) in both cystatin C-based values between proteinuric patients and non-proteinuric patients.

In addition, the stages of CKD in both patients and normal individuals were analysed according to the estimated GFR using the serum creatinine (MDRD) levels. The results revealed that normal eGFR (> 90 mL/min/1.73 m^2^) and serum creatinine levels were reported in stage 1; however, serum cystatin C levels were elevated (1.37 mg/L). Furthermore, serum cystatin C levels were elevated in all stages (stages 1–4) of proteinuric patients compared with serum creatinine (Table 5). The best correlation of UP/UC ratio was noted with the Hoek formula which was 0.635, *P* < 0.001 (Table 6).

## Discussion

The current study was designed to compare the conventional diagnostic method of identifying and monitor CKD patients (proteinuria followed by serum creatinine-based eGFR measurements) over serum cystatin C-based eGFR, an alternative marker. Persistent proteinuria was confirmed by UP/UC which was considered the base-line (2) for proteinuria. The eGFR estimations were conducted based on both serum creatinine and cystatin C (16). Stage 1 CKD patients are the most suitable sample for the present study, but they are rarely present at clinics for CKD in high-prevalent areas. Therefore, a highly susceptible population was screened, and 87 individuals were selected as proteinuric and non-proteinuric for the current study (13).

Only 33% (*n* = 66) of the individuals were confirmed as proteinuric using three early morning urine samples obtained over 3 weeks. Consequently, subjects with normal urinary protein levels in all three samples were identified as normal or healthy. Individuals with two positive results were excluded from the study to increase the accuracy. Proteinuria was confirmed by analysing UP/UC ratio of early morning urine sample of selected individuals. Long-standing diabetes mellitus is one of the risk factor of proteinuria. The proportion of proteinuria among the diabetic patients was suggested in several studies and it varied from 14% to 48%, depending on the population (20–22). However, prevalence of proteinuria among farming communities is rarely identified despite its importance for the prevention of CKDu in the country.

Serum creatinine levels were similar (*P* > 0.05) between the proteinuric and non-proteinuric groups (Table 2). Notably, serum cystatin C level was elevated in the proteinuric group (*P* < 0.05) as compared with the nonproteinuric group. As a marker, it is known that serum creatinine remains less sensitive, and late in detecting kidney disease; false positives may emerge in people who engage in agricultural work in the field (7). In this context, the diagnostic accuracy of serum creatinine and cystatin C was evaluated in terms of sensitivity and specificity, with the UP/UC ratio as the base line. Cystatin C showed excellent diagnostic accuracy (AUC, 0.9675) compared with serum creatinine (AUC, 0.5390) for identifying individuals with proteinuria. The maximum likelihood ratio in ROC analysis (Table 3) was taken as a cut-off value to detect the proteinuric patients. This was 0.93 mg/L for serum cystatin C and 1.015 mg/dL for serum creatinine. Furthermore, both serum creatinine and cystatin C showed significantly positive correlation with UP/UC ratio, and serum cystatin C showed the highest correlation coefficient (Table 6). Hojs et al. observed that cystatin C was an important option, it could be an alternative to serum creatinine for diagnosing and possibly for monitoring kidney function (23). They concluded that, if the purpose of the testing strategy is to monitor the progression of kidney disease in a diagnosed patient, an estimated GFR using serum creatinine, improved through validated equations, is recommended. However, if the purpose of testing is to detect disease in a population at risk for kidney disease, cystatin C-based diagnosis is recommended.

Evidence accumulated over the past years supports the use of cystatin C as an alternative and more sensitive marker for estimation of GFR than serum creatinine and serum creatinine-based GFR estimations (23, 24). Unlike creatinine, cystatin C serum levels are unaffected by age (> 1 year), muscle mass, gender and race. Multiple studies have found cystatin C to be more sensitive to actual changes in GFR in the early stages of CKD than creatinine-based GFR estimates are (25). In fact, large differences in the prevalence of CKD were seen using MDRD and the CG equations in two Japanese studies (10.3% using MDRD, 28.8% using CG) (25, 26).

Pearson’s correlation coefficients with the urine protein to creatinine ratio (UP/UC) showed the most significant correlation with Hoek’s formula (−0.635) in detecting proteinuric patients. Cystatin C-based Larsson equation also showed a significant correlation (−0.592) whereas creatinine based MDRD did not (−0.421). MDRD is the standardised creatinine-based eGFR for categorising patients into CKD stages. This application classified all (100%) healthy (non-proteinuric) individuals into CKD stages 1 and 2. The results show that serum creatinine alone cannot be used for CKD diagnosis in Medawachchiya. All subjects selected for the study were farmers engaged in strenuous agricultural activities under the sun throughout the day time.

## Conclusion

CKDu has been strongly suggested to be associated with proteinuria; thus, the initial diagnosis of the disease was done through proteinuria, followed by serum creatinine eGFR as a functional marker. Serum creatinine-based eGFR did not show any significant difference between proteinuric and non-proteinuric group whereas cystatin C showed a significant reduction of kidney function in proteinuric group. Therefore, it can be concluded that cystatin C-based estimations of kidney function was more accurate for identifying proteinuric patients than creatinine in high risk population for CKDu. However, further studies are recommended to compare the different types of proteinuric patients as diabetic nephropathy apart from the CKDu.

## Figures and Tables

**Table 1:** Distribution of proteinuric status on urinary protein/creatinine (UP/UC) among paddy farmers in Medawatchchia area

Urinary status (*n*)	Gender (*n*)	UP/UC, mean (SD) (mg/mmol)	Age, mean (SD) (years)
Proteinuric (66)	Male (40)	138.1 (12.3)	49 (17)
	Female (26)	120.(10.6)	47 (11)
Non-proteinuric (21)	Male (14)	14.0 (2.3)	45 (9)
	Female (7)	14.5 (2.1)	47 (19)

UP/UC of ≥ 15 mg/mmol and < 15 mg/mmol were considered proteinuric and non-proteinuric (healthy), respectively.

**Table 2:** Serum cystatin C and creatinine concentrations of proteinruric and non-proteinuric subjects as determined by urinary protein to creatinine ratio

	Sex (*n*)	Serum Cystatin C (mg/L) Mean (SD) (range)	Serum creatinine (mg/dL) Mean (SD) (range)
Proteinuric (66)	Male (40)	1.93 (0.52) (0.89–3.0)^a^	1.42 (0.60) (0.65–2.0)
	Female (26)	1.86 ( 0.58) (0.82–2.6)^c^	1.22 (0.30) (0.60–1.8)
Non-proteinuric (21)	Male (14)	0.86 ( 0.30) (0.55–1.22)^b^	1.35 (0.4) (0.90–1.93)
	Female (7)	0.82 (0.25) (0.55–1.04)^d^	1.19(0.4) (0.96–1.72)

a & b, c & d *P* < 0.001 (students *t*-test) between proteinuric and non-proteinuric groups within same sex. Data presented as mean (SD) (range observed)

**Table 3:** Diagnostic performance of serum creatinine and cystatin C (ROC analysis) for identifying proteinuria (UP/UC as base line)

	Creatinine (mg/dL)	Cystatin C (mg/L)
Area under the ROC curve (AUC)	0.5390	0.9675*
Standard Error	0.0655	0.0183
95% confidence interval	0.4104 to 0.6675	0.9316 to 1.003
*P*-value	0.5922	0.0001
Max likelihood ratio	2.12	6.790
Cut-off value	1.015	0.9300

* AUC: −0.9675 (*P* < 0.001)

**Table 4:** Performance of serum creatinine and cystatin C based eGFR estimates (mL/min/1.73 m^2^) on proteinuria as base line

	eGFR-MDRD	eGFR-Hoek	eGFR-Larsson
Proteinuric	63 (24)	46 (16)^a^	44 (17)^c^
Nonproteinuric	68 (16)	101 (28)^b^	109 (37)^d^

a & b *P* = 0.012,

c & d *P* = 0.008 (students *t*-test) between proteinuric (*n* = 66) and non-proteinuric (*n* = 21) individuals. Data presented as mean (SD)

**Table 5:** CKD stage distribution among proteinuric patients (*n* = 66) with reference to serum creatinine

CKD stage	Proteinuric patients *n* (%)	UP/UC (mg/mmol)	eGFR (MDRD) (mL/min/1.73m^2^)	Serum creatinine (mg/dL)	Serum cystatin C (mg/L)
1	10 (15%)	70.0 (4.8)	105 (10)	0.92 (0.16)	1.16 (0.33)
2	24 (37%)	86.0 (2.3)	72 (08)	1.19 (0.21)	1.37 (0.30)
3	30 (45%)	119 (8.2)	43 (08)	1.3 (0.20)	1.90 (0.32)
4	2 (3%)	245 (16)	26 (03)	1.88 (0.38)	2.85 (0.62)

CKD stages 1, 2, 3 and 4 were identified by serum creatinine based eGFR (MDRD equation) to be at > 90, 89–60, 59–30, and 29–15 (mL/min/1.73m^2^), respectively. Stage 5 was not observed.UP/UC of ≥ 15 mg/mmol was considered proteinuric. Data presented as mean + SD unless otherwise stated.

**Table 6:** Association of kidney dysfunction markers with urine protein to creatinine ratio (UP/UC)

	Pearson correlation coefficient
serum creatinine	0.587*
serum cystatin C	0.887*
eGFR-MDRD	−0.421*
eGFR-Hoek	−0.635*
eGFR-Larsson	−0.592*

* *P* < 0.001

## References

[1] Murray CJL, Lopez AD, Mathers CD, Stein C (2001). The Global Burden of Disease 2000 project: aims, methods and data sources. Global Programme on Evidence for Health Policy discussion paper no. 36.

[2] Inker LA, Astor BC, Fox CH, Isakova T, Lash JP, Peralta CA (2012). KDOQI US Commentary on the 2012 KDIGO clinical practice guideline for the evaluation and management of CKD. American Journal of Kidney Diseases.

[3] National Collaborating Centre for Chronic Conditions (UK) (2008). Chronic kidney disease: national clinical guideline for early identification and management in adults in primary and secondary care.

[4] Levey AS, Coresh J, Balk E, Kausz AT, Levin A, Steffes MW (2003). National kidney foundation practice guidelines for chronic kidney disease: evaluation, classification, and stratification. Ann Intern Med.

[5] Roos JF, Doust J, Tett SE, Kirkpatrick CM (2007). Diagnostic accuracy of cystatin C compared to serum creatinine for the estimation of renal dysfunction in adults and children: a meta-analysis. Clin Biochem.

[6] Myers GL, Miller WG, Coresh J, Fleming J, Greenberg N, Greene T (2006). Recommendations for improving serum creatinine measurement: a report from the laboratory working group of the National Kidney Disease Education Program. Clin Chem.

[7] Laterza OF, Price CP, Scott MG (2002). Cystatin C: an improved estimator of glomerular filtration rate?. Clin Chem.

[8] Pucci L, Triscornia S, Lucchesi D, Fotino C, Pellegrini G, Pardini E (2007). Cystatin C and estimates of renal function: searching for a better measure of kidney function in diabetic patients. Clin Chem.

[9] Newman DJ (2002). Cystatin C. Ann Clin Biochem.

[10] Bökenkamp A, Domanetzki M, Zinck R, Schumann G, Byrd D, Brodehl J (1999). Cystatin C serum concentrations underestimate glomerular filtration rate in renal transplant recipients. Clin Chem.

[11] Filler G, Prime F, Lepage N, Sinha P, Vollmer I, Clark H (2002). β-trace protein, cystatin, β_2_-microglobulin, and creatinine compares for detecting impaired glomerular filtration rates in children. Clin Chem.

[12] Takahira R, Yonemura K, Yonekawa O, Iwahara K, Kanno T, Fujise Y (2001). Tryptophan glycoconjugate as a novel marker of renal function. Am J Med.

[13] Jayasekara JMKB, Dissanayake DM, Sivakanesan R, Ranasinghe A, Kumara P, Karunarathna RH (2015). Epidemiology of chronic kidney disease with special emphasis of chronic kidney disease of uncertain etiology in north central region of Sri Lanka. Journal of Epidemiology.

[14] Wanigasuriya KP, Peiris-John RJ, Wickremasinghe R, Hittarage A (2007). Chronic renal failure in North Central Province of Sri Lanka: an environmental exposure induced disease. Trans R Soc Trop Med Hyg.

[15] Jayasekara JMKB, Dissanayake DM, Gunaratne MDN, Thilakarathna S, Sivakanesan R (2013). Agricultural and life style related risk factors of chronic kidney disease of unknown etiology: case control study. International Journal of Medical and Pharmaceutical Sciences.

[16] Athuruliya NT, Abeysekara DT, Amarasinghe PH, Kumarasiri R, Bandara P, Karunarathna U (2011). Uncertain etiology of proteinuric-chronic kidney disease in rural Sri Lanka. Kidney Int.

[17] Wijetunge S, Ratnatunga NVI, Abeysekara TDJ, Wazil AWM, Selvarajah M (2015). Endemic chronic kidney disease of unknown etiology in Sri Lanka: correlation of pathology with clinical stages. Indian Journal of Nephrology.

[18] National Collaborating Centre for Chronic Conditions (UK) (2008). Chronic kidney disease: national clinical guideline for early identification and management in adults in primary and secondary care.

[19] Poge U, Gerhardt T, Stoffel-Wagne B, Klehr HU, Sauerbruch T, Woitas RP (2006). Calculation of glomerular filtration rate based on cystatin C in cirrhotic patients. Nephrol Dial Transplant.

[20] Dreyer G, Hull S, Aitken Z, Chesser A, Yaqoob MM (2009). The effect of ethnicity on the prevalence of diabetes and associated chronic kidney disease. Q J Med.

[21] Wu AY, Tan CB, Eng PH, Tan KT, Lim SC, Tan EK (2006). Microalbuminuria prevalence study in hypertensive patients with type 2 diabetes mellitus in Singapore. Singapore Med J.

[22] Lee WR, Lim HS, Thai AC, Chew WL, Emmanuel S, Goh LG (2001). A window on the current status of diabetes mellitus in Singapore—the Diabcare-Singapore 1998 study. Singapore Med J.

[23] Hojs R, Bevc S, Ekart R, Gorenjak M, Puklavec L (2006). Serum cystatin C as an endogenous marker of renal function in patients with mild to moderate impairment of kidney function. Nephrol Dial Transplant.

[24] Kyhse-Andersen J, Schmidt C, Nordin G, Andersson B, Nilsson-Ehle P, Lindström V (1994). Serum cystatin C, determined by a rapid, automated particle-enhanced turbidimetric method, is a better marker than serum creatinine for glomerular filtration rate. Clin Chem.

[25] Konta T, Hao Z, Abiko H, Ishikawa M, Takahashi T, Ikeda A (2006). Prevalence and risk factor analysis of microalbuminuria in Japanese general population: the Takahata study. Kidney Int.

[26] Ninomiya T, Kiyohara Y, Kubo M, Tanizaki Y, Doi Y, Okubo K (2005). Chronic kidney disease and cardiovascular disease in a general Japanese population: the Hisayama study. Kidney Int.

